# Reactive Oxygen and Nitrogen Species in Plants―2nd Edition

**DOI:** 10.3390/antiox15060777

**Published:** 2026-06-22

**Authors:** Francisco J. Corpas, Rosa M. Rivero, José M. Palma

**Affiliations:** 1 Group of Antioxidants, Free Radicals and Nitric Oxide in Biotechnology, Food and Agriculture, Department of Stress, Development and Signaling in Plants, Estación Experimental del Zaidín, Spanish National Research Council (CSIC), C/Profesor Albareda 1, 18008 Granada, Spain; josemanuel.palma@eez.csic.es; 2 Department of Plant Nutrition, Centro de Edafología y Biología Aplicada del Segura (CEBAS) CSIC, Campus Universitario de Espinado, EDF. 25, 30100 Murcia, Spain; rmrivero@cebas.csic.es

Although hydrogen peroxide (H_2_O_2_) and nitric oxide (NO) are widely recognized for their signaling functions [1,2], they represent broader families of reactive oxygen (ROS) and nitrogen (RNS) species that exhibit an even wider range of physiological actions [3,4,5]. Accumulating data show that these molecules participate in numerous plant physiological processes, including seed and pollen germination [6,7], the development and regulation of root architecture [8,9], photosynthesis [10], stomatal movement [11], senescence [12], flowering [13], and fruit formation and ripening [14,15], but also in the mechanism of response against biotic and abiotic stresses [16,17,18,19]. Figure 1 summarizes the landscape of processes involving the participation of ROS and RNS in higher plants.

This second edition of the Special Issue on ROS and RNS in plants includes three research articles and two reviews, which extend our knowledge in different aspects of the implications of these reactive molecules in different plant organs and species under physiological and biotic and abiotic stresses.

In the manuscript by Ramírez-Mellado et al. [20], the number of genes that encode the antioxidant enzyme peroxirredoxin (PRX) in pepper fruits and how its expression is modulated during ripening and in an enriched atmosphere of NO is analyzed. Among the eight identified *PRX* genes, it was found that, during ripening, *CaPRX1* and *CaPRX2E* were upregulated, *CaPRX2B* and *PRX* Q were downregulated, and the other three genes showed no significant changes. When sweet pepper fruits were treated with NO gas, *CaPRX2B* and *CaPRXQ* increased in expression, while *CaPRX1* and *CaPRX2*-*CysBAS1* decreased. Additionally, four PRX proteins were identified as targets of *S*-nitrosation, a NO-derived post-translational modification that can affect protein function. Therefore, this study demonstrates that *PRX* genes in pepper fruits are regulated differently during ripening and by exogenous NO, both at the gene and protein levels. These findings highlight the important role of Prxs in the fruit’s antioxidant defense system, helping peppers cope with nitro-oxidative stress during ripening.

In the study by Tolrà et al. [21], the effects of halopriming with NaCl were evaluated in seeds of the halophyte *Cakile maritima*. Halopriming was proposed to induce a form of stress “memory” that enables a faster and more efficient response when seedlings are subsequently exposed to the same stressor. The results showed that halopriming promoted adaptive responses, improving both seed germination rates and biomass production. Furthermore, the oxidative stress induced by NaCl was alleviated, as halopriming positively modulated several antioxidant enzymes such as catalase and superoxide dismutase, resulting in lower levels of lipid peroxidation and reduced H_2_O_2_ accumulation. Additionally, the effects of halopriming appear to be mediated by genes involved in Na^+^/H^+^ exchange, such as *NHX1* and *Salt Overly Sensitive 1* (*SOS1*), as well as by the transcription factor WRKY25, which has previously been shown to mediate oxidative stress tolerance in *Arabidopsis thaliana* [22].

Using rice (*Oryza sativa*) exposed to the fungus *Magnaporthe oryzae*, Wang et al. [23] demonstrated the critical role of the Mitogen-Activated Protein (MAP) kinase signaling component OsMEK2 in regulating ferroptotic cell death as part of rice immune responses against this pathogen. These authors showed that OsMEK2 positively controls Ca^2+^-mediated signaling, promoting iron and ROS accumulation, lipid peroxidation, and hypersensitive cell death—key defense mechanisms that restrict fungus pathogen invasion. Thus, the loss of OsMEK2 function impaired these responses, resulting in reduced immune-associated ferroptosis and increased susceptibility to infection. Mechanistically, OsMEK2 appears to regulate the expression of genes involved in ROS production and Ca^2+^ signaling, which encode for the NADP-dependent malic enzyme (NADP-ME), the superoxide-generating Ca^2+^-dependent Respiratory Burst Oxidase Homolog B (RBOHB), phospholipase C (OsPLC), and cyclic nucleotide-gated channels (OsCNGCs). These findings are particularly relevant because they uncover a previously unrecognized link between MAPK signaling, Ca^2+^ dynamics, and ferroptotic cell death in plant immunity, providing new insights into how plants coordinate redox and ion signaling to support effective defense responses. Thus, the authors suggest that OsMEK2 is a potential molecular target for improving disease resistance in rice and possibly other crops.

The review by Anee et al. [24] highlights the central role of ROS and NO in coordinating plant responses to combined abiotic stresses, particularly salt stress and waterlogging, which are increasingly prevalent under climate change conditions. Although ROS can cause cellular damage when accumulated excessively, they also function as essential signaling molecules that interact with NO, Ca^2+^, protein kinases, ion transport systems, and phytohormones to activate adaptive responses. The review emphasizes the importance of chloroplasts and mitochondria as key sources of stress-induced ROS and as critical hubs for energy regulation and stress signaling. Importantly, it suggests that plant responses to combined stresses are not merely the sum of individual stress responses, but involve specific and finely coordinated signaling networks uniquely activated under simultaneous stress conditions. Within these networks, the interplay between ROS and NO appears to act as a central regulatory mechanism, integrating multiple signaling pathways to optimize plant acclimation and resilience. This overview is particularly relevant because it advances our understanding of how redox signaling contributes to stress adaptation in complex environments, offering valuable insights for the development of crops with improved tolerance to multiple, overlapping environmental challenges.

On the other hand, the review article by Liu et al. [25] focuses in the signaling properties of H_2_O_2_, highlighting its key role in the regulation of root apical meristem (RAM) activity, a critical process for root growth, development, and environmental adaptation. Beyond its traditional recognition as a ROS, H_2_O_2_ is presented as an essential regulator of the quiescent center (QC) and stem cell niche (SCN), both of which are fundamental for maintaining root meristem function and plasticity. The authors emphasize that H_2_O_2_ modulates auxin distribution by influencing intercellular transport, thereby affecting the auxin gradients required for RAM maintenance. Additionally, H_2_O_2_ regulates the expression of major developmental factors, including WOX5, PLETHORA (PLT), SHORTROOT (SHR), and SCARECROW (SCR), integrating redox signaling with core genetic pathways controlling root stem cell identity and activity. The relevance of this work lies in demonstrating how H_2_O_2_ acts as a central hub linking developmental regulation and environmental responsiveness, advancing our understanding of ROS signaling in root biology and highlighting its importance in plant adaptation to changing environmental conditions.

Finally, we would like to express our sincere gratitude to all of the authors who have shared their valuable research in this Special Issue, providing important insights into recent advances in the understanding of the roles of ROS and RNS in plants. Their work collectively highlights the growing complexity and relevance of redox regulation in plant biochemistry and physiology. We also extend our appreciation to the readers and researchers whose continued interest in this field helps drive scientific discussion and future discoveries in plant stress biology and signaling.

## Figures and Tables

**Figure 1:**
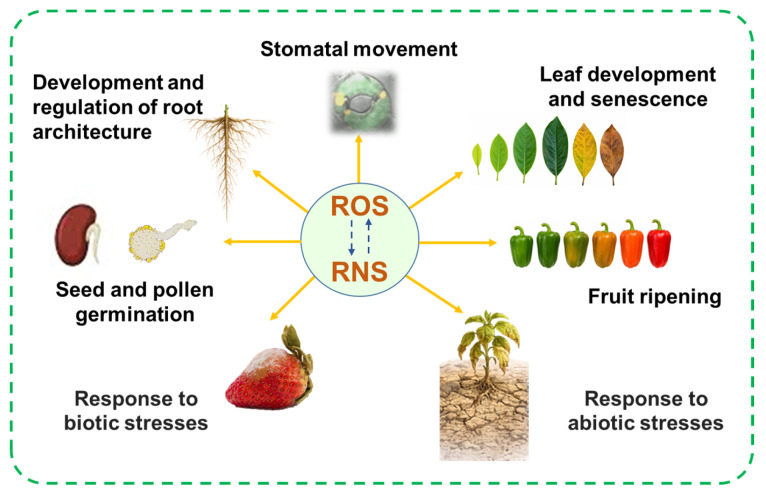
Schematic overview of ROS and RNS participation in plant physiological processes and signaling pathways triggered by biotic and abiotic stresses.
